# Assessing critical temperature dose areas in the kidney by magnetic resonance imaging thermometry in an ex vivo Holmium:YAG laser lithotripsy model

**DOI:** 10.1007/s00345-022-04255-1

**Published:** 2022-12-21

**Authors:** Robert Wriedt, Mehmet Yilmaz, Thomas Lottner, Andreas Reichert, Konrad Wilhelm, Philippe-Fabian Pohlmann, Christian Gratzke, Michael Bock, Arkadiusz Miernik

**Affiliations:** 1 Department of Urology, University of Freiburg-Medical Center, Faculty of Medicine, Hugstetter Str. 55, 79106 Freiburg, Germany; 2 Department of Ophthalmology, University Medical Center Hamburg-Eppendorf (UKE), Martinistr. 52, 20246 Hamburg, Germany; 3 Department of Radiology, Medical Physics, University of Freiburg-Medical Center, Faculty of Medicine, Freiburg, Germany

**Keywords:** Ex vivo, Holmium laser, Kidney, Lithotripsy, Temperature development

## Abstract

**Purpose:**

We aimed to assess critical temperature areas in the kidney parenchyma using magnetic resonance thermometry (MRT) in an ex vivo Holmium:YAG laser lithotripsy model.

**Methods:**

Thermal effects of Ho:YAG laser irradiation of 14 W and 30 W were investigated in the calyx and renal pelvis of an ex vivo kidney with different laser application times (*t*_*L*_) followed by a delay time (*t*_*D*_) of *t*_*L*_/*t*_*D*_ = 5/5 s, 5/10 s, 10/5 s, 10/10 s, and 20/0 s, with irrigation rates of 10, 30, 50, 70, and 100 ml/min. Using MRT, the size of the area was determined in which the thermal dose as measured by the Cumulative Equivalent Minutes (CEM_43_) method exceeded a value of 120 min.

**Results:**

In the calyx, CEM_43_ never exceeded 120 min for flow rates ≥ 70 ml/min at 14 W, and longer *t*_*L*_ (10 s vs. 5 s) lead to exponentially lower thermal affection of tissue (3.6 vs. 21.9 mm^2^). Similarly at 30 W and ≥ 70 ml/min CEM_43_ was below 120 min. Interestingly, at irrigation rates of 10 ml/min, *t*_*L*_ = 10 s and *t*_*D*_ = 10 s CEM_43_ were observed > 120 min in an area of 84.4 mm^2^ and 49.1 mm^2^ at *t*_*D*_ = 5 s. Here, *t*_*L*_ = 5 s revealed relevant thermal affection of 29.1 mm^2^ at 10 ml/min.

**Conclusion:**

We demonstrate that critical temperature dose areas in the kidney parenchyma were associated with high laser power and application times, a low irrigation rate, and anatomical volume of the targeted calyx.

**Supplementary Information:**

The online version contains supplementary material available at 10.1007/s00345-022-04255-1.

## Introduction

Holmium:yttrium–aluminium-garnet (Ho:YAG) laser lithotripsy has been the most widely used, effective, and safe minimally invasive surgical method in the last two decades and is the gold standard for treating urinary tract stones according to the European Association of Urology (EAU) guidelines [1, 2]. It is well known that stone ablation during laser lithotripsy is caused by photothermal and photoacoustic effects [3, 4]; due to the excellent energy absorption characteristics in the water of a Ho:YAG laser, a temperature rise is expected that can have an impact on the surrounding tissue [5].

Although there are many in vitro [6–10] and in vivo [11–13] studies investigating the temperature development during Ho:YAG laser lithotripsy, knowledge about the spatially resolved temperature development during lithotripsy is limited [14]. For instance, temperature probes near the laser only reveal local thermal reactions in the parenchyma, and temperature measurements of the irrigation fluid reflect the physical conditions only indirectly. So far, different types of local thermocouples have been used for temperature measurement [6, 7, 14, 15], and a different approach is required to assess the temperature distribution in the kidney during laser application.

An alternative to pointwise temperature measurements is magnetic resonance imaging thermometry (MRT) which provides quantitative images of the temperature change by measuring the temperature-induced change in proton resonance frequency [16]. During interventions, MRT is used in the evaluation, post-procedural follow-up, and monitoring of laser thermotherapy and different kinds of ablation [17–20]. To provide a precise quantification of the thermal energy distribution in the renal parenchyma during Ho:YAG laser treatment, we evaluated in an ex vivo Ho:YAG laser lithotripsy model the temperature dose in the kidney using an MRT.

## Materials and methods

### Experimental setup

To systematically evaluate the dependency of tissue heating on various lithotripsy parameters, an ex vivo porcine kidney model was developed. Porcine kidneys were fixed in a plastic ring which was suspended in a water bath heated to a body temperature of 37 °C (Fig. 1A- Online Resource 1). To maintain this temperature, a thermostat (Eheim GmbH & Co. KG, Deizisau, Germany) heated 5 l of tap water to 45 °C in a polyethylene container. A hose pump (SP04L, Samed GmbH, Dresden, Germany) continuously conducted heated water through the water bath via a closed tubing system. Irrigation was provided by a Reglo-Z Digital pump (Cole Parmer, Chicago, USA). To be able to compare the irrigation settings, the kidneys were flushed with 22.1 °C tap water at flow rates of 100, 70, 50, 30, and 10 ml/min. Before starting the experiment, the kidneys were rinsed for at least 30 s to homogenize the temperature conditions in the kidneys. As the ureterorenoscopy (URS) model, we used 6 and 10 Fr-sized plastic bougie dilators, into which irrigation fluid and laser fiber were inserted (Fig. 1B- Online Resource 1). The laser fiber, access for the irrigation fluid, and fiber-optic temperature probes (FOTP) were fed through a plastic straw with a diameter of 5 mm. A RigiFib laser fiber (550 μm) and a Sphinx Junior 30 W Ho:YAG laser (LISA, Kaltenburg-Lindau, Germany) were used. The kidney unit was replaced after the test series with 100 ml/min irrigation and all laser settings. The experimental setup is shown in Fig. 1C (Online Resource 1).

### Laser application

The URS model was initially positioned in the upper calyx, and laser irradiation was performed with two parameter settings: 14 W with 1.2 J, 12 Hz, 90 μs pulse duration, and 30 W with 3.3 J, 9 Hz, 230 μs pulse duration. Later, the laser fiber was positioned in the renal pelvis where all experiments were carried out with a single power level of 30 W. To simulate a realistic lithotripsy setting, the duration of the laser application *t*_*L*_ was reduced to 5 or 10 s, applied three times successively with an intermittent delay of *t*_*D*_. The following application protocols were applied: *t*_*L*_/*t*_*D*_ = 5/5 s, 5/10 s, 10/5 s, 10/10 s, and 20/0 s. In the calyx at *t*_*L*_/*t*_*D*_ = 5/5 s and 30 W the laser was applied four times to assess the temperature increase with more frequent laser applications. In a further experiment, human kidney stones from previous interventions in our department were positioned in the calyx, and the laser was applied at 14 W, *t*_*L*_ = 20 s, and a flow rate of 30 ml/min. After the experiments, the kidney was cut open and assessed for macroscopically visible pathologies at the laser exposure’s location.

### Temperature measurements

Temperature measurements were carried out in a clinical 1.5 T MRI system (Tim Symphony, Siemens, Erlangen, Germany) using an anterior flex loop coil and the superior integrated spine coils for signal reception. Images were acquired dynamically before, during, and after laser lithotripsy using the proton resonance frequency (PRF) method. Therefore, data were acquired with a segmented echo planar imaging (EPI) pulse sequence with the following parameters: repetition time TR = 31 ms, echo time TE = 15 ms, flip angle α = 13°, slice thickness SL = 4 mm, imaging field-of-view: 235 × 259 mm^2^, matrix = 174 × 192 resulting in an area per pixel of 1.82 mm^2^ and an acquisition time per image of TA = 620 ms. From the dynamic data, temperature difference maps were calculated [16], and critical temperature areas were determined (see below). For reference, one FOTP (FOTEMP 6–19, Optocon AG, Dresden, Germany) was positioned in the straw at a 6 cm distance from the tip of the laser fiber, which guides the irrigation fluid from the renal pelvis. One FOTP was placed in the pool water to control the constant temperature, another was fixed in the renal parenchyma.

### Data analysis

The dynamic PRF images acquired during each experiment were stored as DICOM images. Using in-house software developed in MATLAB, vR2020a (MathWorks, Massachusetts, USA), the temperature difference from the baseline was calculated and displayed for each individual pixel. Image pixels contaminated with motion artifacts caused by flushing, turbulence, or phase noise due to low signal-to-noise ratio were excluded from data analysis. For this purpose, all pixels with an MRI signal magnitude of less than 3.7 times that of the mean signal in the air outside the water bath (i.e., *S* < 3.7·*S*_*air*_) were considered as noise and were therefore removed (Fig. 2A- Online Resource 2). Furthermore, a Region of Interest (ROI) was drawn manually in the kidney, and only pixels in the ROI (without the ureter) were included in the analysis to exclude flow-related areas from outside of the kidney (Fig. 2B- Online Resource 2). To reduce the effect of phase noise, an averaging filter with a width of 3 × 3 pixels was applied.

To assess the local thermal dose delivered in each experiment, the Cumulative Equivalent Minutes (CEM_43_) method as described by Sapareto and Dewey [21] was used to calculate dose maps from the dynamic temperature data. Note, that CEM_43_ indicates the time an elevated temperature needs to be applied to cause the same thermal damage as a temperature of 43 °C [22]. Since there is evidence that > 120 min causes thermal damage in most tissues including renal parenchyma [21, 22], this was considered as the critical temperature threshold in this study. After calculation of the CEM_43_ in each pixel, the area of all image pixels was summed up which exceeded 120 min. Furthermore, the pixel showing the highest temperature difference from the temperature values of all included pixels was selected automatically (Fig. 3- Online Resource 3). These results were analyzed using GraphPad Prismv9 (GraphPad, San Diego, USA).

## Results

A total of 325 experiments were conducted, 260 of them with intermittent laser application, and 51 with *t*_*L*_ = 20 s. Four of the experiments were excluded as air suction into the renal pelvis through the tubing resulted in image artifacts that rendered the temperature curves unevaluable. The FOTP inserted into the parenchyma showed no increase in temperature in any of the experiments. The sample images of MRI thermometry during the experiments are shown in Fig. 3 (Online Resource 3).

### Areas of critical temperature doses

#### Calyx

The MRI-based calculated volume of the renal calyx averaged 0.95 cm^3^ (0.83–1.04 cm^3^). Table 1 summarizes the areas with CEM_43_ > 120 min in the calyx and the renal pelvis as a function of the applied laser power and the irrigation rate (Online Resource 4). At high irrigation rates (*I*) of *I* ≥ 50 ml/min, CEM_43_ remained below the threshold of 120 min at any setting except for *t*_*L*_/*t*_*D*_ = 10/10 s, where a small area of 2.2 mm^2^ was found. We observed that extending the delays between laser applications by a further 5 s (*t*_*D*_ = 10 s) led to smaller areas of damage (Fig. 4A- Online Resource 5). At 30 W in the calyx, *t*_*L*_/*t*_*D*_ = 5/5 s led to a distortion of the representation, since in this test series the laser was used four instead of three times. At ≥ 30 ml/min, the areas were larger with *t*_*L*_ = 10 s than *t*_*L*_ = 5 s. The irrigation rates of ≥ 70 ml/min did not result in any excessive dose, with one exception, a one-time irrigation rate of 100 ml/min resulted in calculated damage of 7.3 mm^2^ for *t*_*L*_/*t*_*D*_ = 5/10 s. At 10 ml/min, it was found that the areas with *t*_*L*_*/t*_*D*_ = 10/10 s were significantly larger than those with *t*_*L*_*/t*_*D*_ = 10/5 s (84.4 vs. 49.1 mm^2^) (Fig. 4B- Online Resource 5).

#### Renal pelvis

The volume of the renal pelvis in the MR images was 8.86 cm^3^ (7.73–10.88 cm^3^). Figure 4C shows the area size with intermittent laser application with a power of 30 W when the laser fiber is positioned in the renal pelvis (Online Resource 5). Short laser intervals (*t*_*L*_ = 5 s) only showed endangered areas at 10 ml/min with a calculated maximum of 29.1 mm^2^. At 10 ml/min, we observed increased temperature doses in all the laser applications. In a direct comparison, longer breaks between laser treatments resulted in smaller areas than shorter breaks. At 70 ml/min, an increase up to 10.9 mm^2^ in CEM_43_ > 120 min areas was seen in all tests included in the evaluation.

#### Additional experiments

In an additional measurement, a delay setting of *t*_*L*_*/t*_*D*_ = 20/0 s was also tested. A fiber location in the pelvis resulted in smaller doses inside a renal calyx—even at irrigation rates of 10 ml/min, a maximum CEM_43_ of 120 min was exceeded in an area of 18 mm^2^. With a calyceal fiber positioning at 14 W and *I* = 10 ml/min, we detected areas measuring 57 mm^2^. Irrigation rates of 50 ml/min resulted in smaller areas of a maximum of 16 mm^2^. The values at 70 ml/min yielded an area of 84 mm^2^ which can be attributed to the fact that these experiments were carried out after the organ was replaced. Here, the fiber was positioned very close to the parenchyma, and the kidney tissue was burnt over a large area, which was later confirmed macroscopically. At 30 W with calyceal fiber, we noted a course that depended on the flushing rate. That area measured more than 30 mm^2^ at ≥ 50 ml/min (Fig. 4D- Online Resource 5).

#### Measurements with stone material

In the measurements with inserted stone material, we identified a temperature increase before the laser application began, most likely due to artifact by moving stone material. After we stopped using the laser, the temperature decreased but rose again from 56th second (Fig. 5A- Online Resource 6).

After the experiment, brownish burnt areas appeared macroscopically in each of the test organs in the immediate vicinity of the fiber layer, which also ran a few millimeters into the parenchyma (Fig. 5B- Online Resource 6).

## Discussion

In recent years, a number of studies have increased that investigate the temperature formation during laser lithotripsy and its effects on the tissue. However, there is still too little evidence on actual thermal conditions in the kidney, especially in renal parenchyma. In the present study, we use MRI thermometry, which provides spatially resolved temperature measurements, to calculate critical temperature dose areas in the kidney parenchyma in an ex vivo model during laser lithotripsy. In line with previous publications, we identified the laser power, laser application time, irrigation rate, and calyx volume to be those factors influencing thermal effects on the renal parenchyma.

Several studies have shown that higher irrigation rates are required to ensure patient safety during laser lithotripsy [8, 23]. Hein et al. reported that the temperature decreases rapidly as the irrigation rates increase [8]. Maxwell et al. found that while tissue injury was observed at 15 ml/min with a power of 40 W, there was no dangerous temperature increase at 40 ml/min [24]. In accordance with their investigations, we observed that the irrigation rate should increase as the laser power increases to ensure tissue safety. We found that in kidney calyces an irrigation rate of 50 ml/min was sufficient at 14 W, and 70 ml/min was required at 30 W to adequately remove the thermal energy. Note that the URS model used in our study constricts the infundibulum of the calyx, which could make the circulation of the irrigation fluid more difficult [9, 25]. In addition, the general reduction in fluid circulation within small cavities might explain higher temperature values in calyces.

The volume of the calyx has a significant influence on a temperature rise during laser lithotripsy [8, 24, 26]. Rezekahn et al. reported on the influence of laser energy on variously sized glass bulbs between 0.5 and 60.8 ml, when laser energy was applied 1 min continuosly at an irrigation rate of 40 ml/min. Under these circumstances, temperatures could be measured below the tissue-damaging threshold [23]. Their group showed that smaller cavities lead to more hazardous temperature development in the kidney. Presumably, due to its large and smooth surface, the glass bulb might impact thermal conductivity to the surrounding water bath; however, this factor is to be considered rather inferior since the thermal conductivity of glass is assumed to be poor. Furthermore, the time to reach a plateau phase after the temperature rise was inversely proportional to the cup volume [26]. The calyx size in the present study averaged 0.95 cm^3^. Therefore, the in vitro study of Aldoukhi et al. with a glass bulb volume of 0.7 ml is the most suitable study to be compared with the present investigation [6]. Aldoukhi et al. concluded that there is no thermal hazard to the bowl at 15 W at an irrigation rate of at least 10 ml/min. At 30 W, they recommended an irrigation rate of minimally 20 ml/min. These values are below the thresholds we determined in this study, which were recorded at an irrigation rate of 30 ml/min, provided the laser is not continuously used for longer than 5 s at 14 W. At 30 W, no values greater than 120 min CEM_43_ were achieved at an irrigation rate of 70 ml/min at the lowest.

In practice, only three concomitant energy applications during laser lithotripsy, as in this study, might not be sufficient to fragment the stones. At four applications in the calyx with *t*_*L*_* / t*_*D*_ = 5/5 at 30 W, we noted high CEM_43_ areas averaging 88 mm^2^ at a low flushing rate. In contrast to higher irrigation rates, low irrigation rates of 10 and 30 ml/min were associated with a step-like temperature rise at maximum temperatures per laser application. This increase affects not only the peak values during the laser application but also the peak values at the pause, provided the irrigation rate was ≤ 50 ml/min. If the temperature no longer decreases sufficiently during the pauses, the temperature increases overall applications causing tissue damage even during the pausing phases. Aldoukhi et al. found no temperature exceeding 51 °C when applying 40 W for 10 s in a glass tube model without rinsing [7]. They concluded that a *t*_*L*_ = 10 s protects the patient from inadvertent temperature peaks. However, in the present study, we found that with a laser application of 30 W for 10 s in the renal calyx, an irrigation rate of at least 70 ml/min is necessary to avoid exceeding 120 min CEM_43_. This difference might be attributed to the different laser powers and materials in our experiments.

In the experiments with human stones, we observed a strong focal temperature development on the calyx wall. One reason for this hot spot could be that after the stone fragmentation the laser fiber tip is located between the stone fragments, and the heat generated may affect the parenchyma more directly since the stone is no longer acting as a barrier. Another reason for focal temperature development in the calyx could be that since the fragmented stone pieces heated by laser energy during lithotripsy are not removed from the surgical area in the kidney and the irrigation fluid’s cooling effect has not yet started, the heat emitted from the fragmented stone pieces may cause a temperature rise in parenchymal tissue. Furthermore, artifacts due to gas creation or stone movement should be considered in this experimental setup.

Regarding practical considerations, the results of our study can be interpreted as relevant for flexible URS (fURS). Hein et al. have shown that hazardous temperature values can occur in the presence of inadequate irrigation even at 5 W power laser power during an fURS procedure [14]. In another study, it was reported that when irrigation was stopped during fURS, the temperature rapidly reached 43 °C [27]. Therefore, it is crucial to provide continuous irrigation during laser application within the urinary tract. In addition, close monitoring of irrigation during intrarenal laser application and ensuring its continuity is vital for patient safety. From the surgical perspective, there may be situations where irrigation needs to be interrupted. Under such circumstances, continuous laser usage should be applied carefully and be kept to a minimum. Furthermore, various ureteral access sheaths in combination with different scopes might severely affect the irrigation fluid flow. Therfore, the ureteral access sheath must be placed correctly and fluid outflow needs to be regularly controlled during the surgery.

The study has limitations to acknowledge. Only two standard laser settings were tested, and the pulse duration’s impact was not investigated. Because of the MRI device’s strong magnetic field, this experimental setup had to be designed with instruments without ferromagnetic materials. Therefore, the URS model we developed only partially corresponds to the instruments used in clinical practice. Due to time constraints, temperatures were only measured in a 2D slice defined by the MRI experiment, and no temperature information was obtained above and below this slice. This additional information could be obtained using advanced MRI techniques with orthogonal image slice [28]; however, this could only be achieved with a lower temporal resolution during the dynamic measurements. Furthermore, the irrigation characteristics and rates in the experiments might be considered biased since we tested only continuous flushing, and no blood perfusion of the kidney was present. Perfused kidney tissue hinders the temperature rise as the heated blood is continuously removed from the kidney, thereby achieving a cooling effect [29]. However, this effect might not be of great clinical importance, as Khoder et al. demonstrated that laser-damaged zones are similar in perfused and non-perfused porcine kidneys [30]. Because of the lack of optical control while applying laser energy, we could not detect any potential displacement of the laser fiber, which may have caused additional hot spots in the tissue. All tests were repeated three to eight times which is not sufficient to achieve statistically robust results. However, our results may suffice to reveal tendencies in temperature development during Holmium laser lithotripsy. In addition, with an almost unlimited number of combinations involving single pulse energy, frequency, pulse duration, the pattern of energy application, and irrigation rates it is virtually impossible to measure all combinations sufficiently. Here, only thermal simulations might help to identify additional parameters for heat transport and tissue damage.

Accidental movements in the experimental setup could have led to incorrect image processing. The macroscopic specimen inspection after the experiment was semi-quantitative and semi-qualitative. It was not possible to assign the macroscopic damage to individual tests, since, for practical reasons, the specimens could not be replaced after each test run. An assessment according to histological criteria was not possible, since the kidneys were examined postmortem and after deep de-freezing. Finally, our CEM_43_ model might not reflect genuine thermal damage because repair mechanisms and individual impacts on the organism are not fully understood. It is rather a surrogate parameter reflecting thermal stress to cells in vivo, for example, which is used to assess thermal tissue damage for example during hyperthermal therapy of liver metastases.

## Conclusions

The present results show relevant thresholds for a Holmium laser lithotripsy setting concerning the intraparenchymal formation of critical temperature areas. We have shown that the laser energy, application time, irrigation rate, and calyx volume influence the potential areas of tissue damage in the kidney as measured by CEM_43_ values. Our results deliver supporting evidence that laser lithotripsy can lead to dangerous temperature developments. Using MRI, the measurements show for the first time the spatially resolved distribution of thermal energy in the renal parenchyma. These data should sensitize the urologist to possible damage to the urinary tract while carrying out laser-based stone therapy.

## Supplementary Information

Below is the link to the electronic supplementary material.

Supplementary file1 Fig.1: A) View of the experiment tank from above. The organ is fixed with two rubber bands in a blue plastic ring and on a plastic grid. The ureter leads to a sluice, through which a straw (orange) with a laser fiber, laser fiber guide rail, and irrigation fluid is fed. B) URS model: A straw (orange) contains a 6 Fr light blue bougie dilator to guide the irrigation fluid and a 10 Fr dark blue bougie dilator to guide the laser fiber. C) Schematic representation of the experimental setup: a: FOTEMP 6-19 for temperature monitoring using FOTP.b: Laser Sphinx Jr. c: Irrigation fluid container. d: Reglo Z Digital rinsing pump for sucking in the rinsing liquid. e: Heating pool with thermostatic heater. f: Hose pump Samed SP04L. g: Test tank with kidney and URS model. (PDF 513 KB)

Supplementary file2 Fig.2: A) Drawing an ROI (green) to determine the background noise in the area of air. B) Drawing an ROI (red) that includes only the renal parenchyma and renal pelvis. (PDF 146 KB)

Supplementary file3 Fig.3: A) Anatomical MR image (in gray) with superimposed temperature map (in glow scale) with a position of the fiber in the pelvis and an irrigation rate of 70 ml/min. At the tip of the laser fiber heating occurred at the edge of the renal pelvis near the hilum (green arrow). B) Lithotripsy with 14 W, tL=20 s and a flushing rate of 30 ml/min of stone granules in the calix before (left) and after (right) start of the laser application. C) Repeated lithotripsy with the same parameters as in B). (PDF 120 KB)

Supplementary file4 Table 1: Mean area of the pixels with CEM43> 120 min according to applied laser power and the irrigation rate. (PDF 42 KB)

Supplementary file5 Fig.4: Areas with CEM43> 120 min with different laser fiber positions and powers: A) 14 W [1.2 J, 12 Hz] and a fiber located in the renal calyx. B) 30 W [3.3 J, 9 Hz] and fiber located in the renal calyx. C) 30 W and fiber located in the renal pelvis. D) With tL=20 s. The mean values and the standard deviation of the respective test series are plotted in black. (PDF 240 KB)

Supplementary file6 Fig.5: A) Temperature curve during tL/tD=20/0 s with 14 W on stone granules in the kidney calyx and 30 ml/min flushing rate. The phase of the laser application is highlighted in red. B) Macroscopic view of the thermal lesion -tissue injury is clearly visible as a brown and dark area in the urothelium and the parenchyma. (PDF 204 KB)

## Data Availability

The raw data are with the corresponding author and can be provided on request.

## Code availability

Not applicable for this section.
