# Supplementary figures and images for: Assessing critical temperature dose areas in the kidney by magnetic resonance imaging thermometry in an ex vivo Holmium:YAG laser lithotripsy model
Source: World J Urol. 2022 Dec 21;41(2):543–9. doi: 10.1007/s00345-022-04255-1 (PMC9947089; doi:10.1007/s00345-022-04255-1)

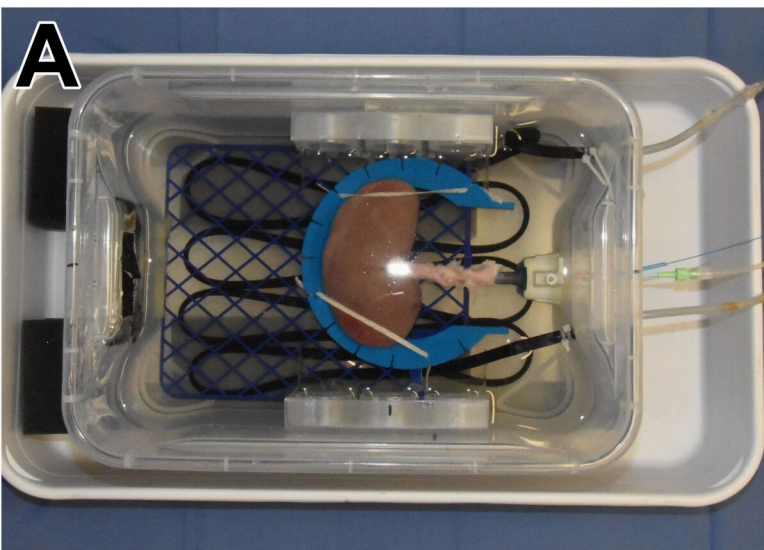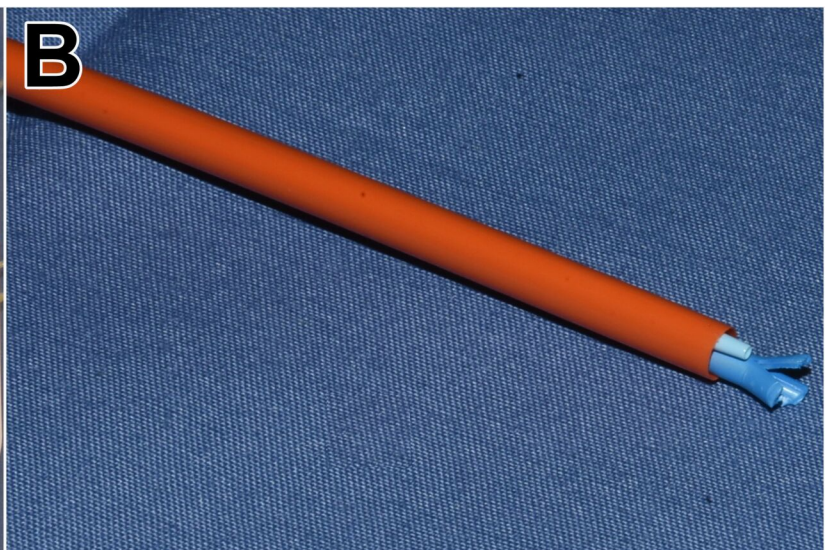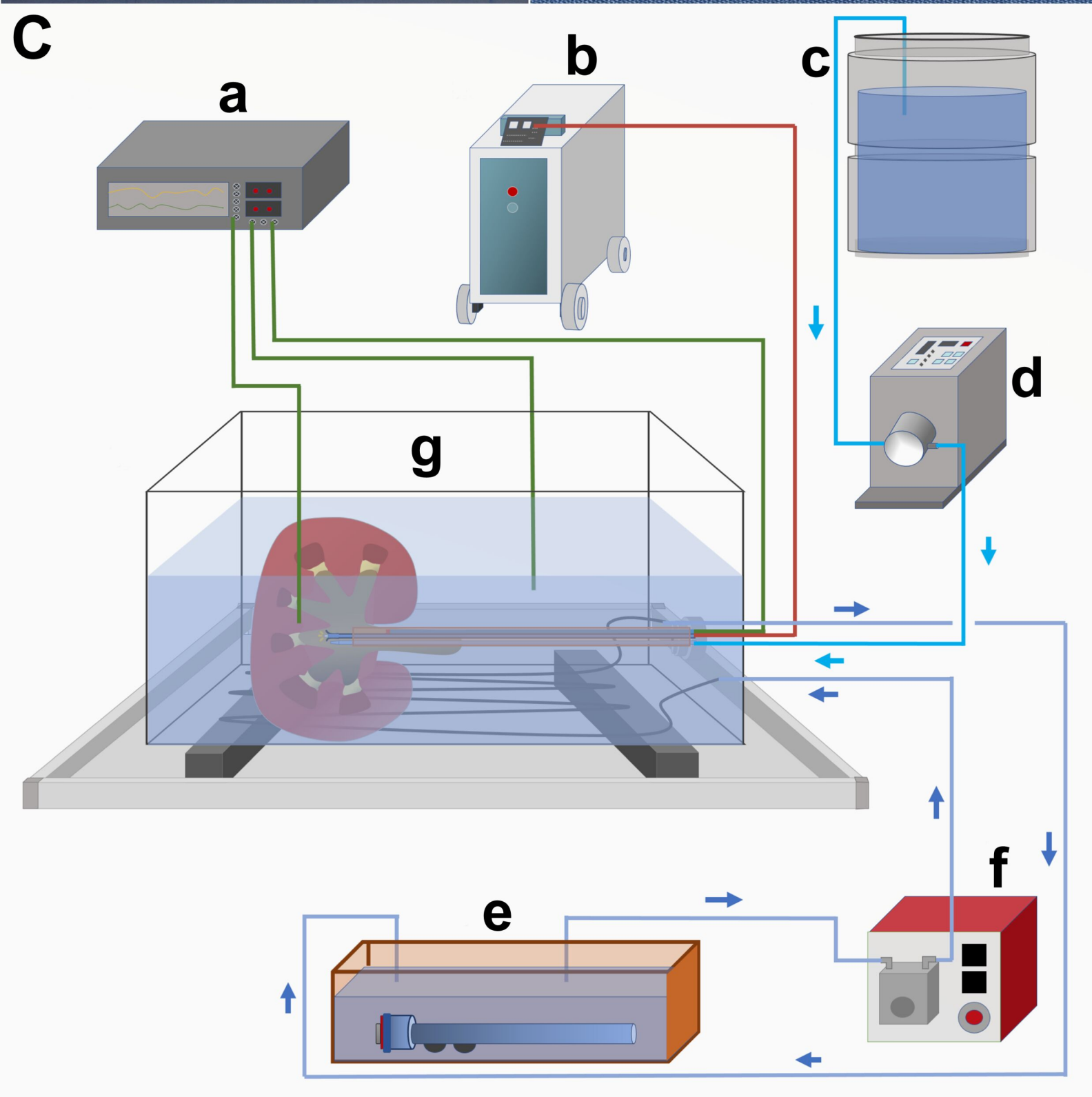

Supplement: Supplementary file 1 — Supplementary file1 Fig.1: A) View of the experiment tank from above. The organ is fixed with two rubber bands in a blue plastic ring and on a plastic grid. The ureter leads to a sluice, through which a straw (orange) with a laser fiber, laser fiber guide rail, and irrigation fluid is fed. B) URS model: A straw (orange) contains a 6 Fr light blue bougie dilator to guide the irrigation fluid and a 10 Fr dark blue bougie dilator to guide the laser fiber. C) Schematic representation of the experimental setup: a: FOTEMP 6-19 for temperature monitoring using FOTP.b: Laser Sphinx Jr. c: Irrigation fluid container. d: Reglo Z Digital rinsing pump for sucking in the rinsing liquid. e: Heating pool with thermostatic heater. f: Hose pump Samed SP04L. g: Test tank with kidney and URS model. (PDF 513 KB) [file 345_2022_4255_MOESM1_ESM.pdf]

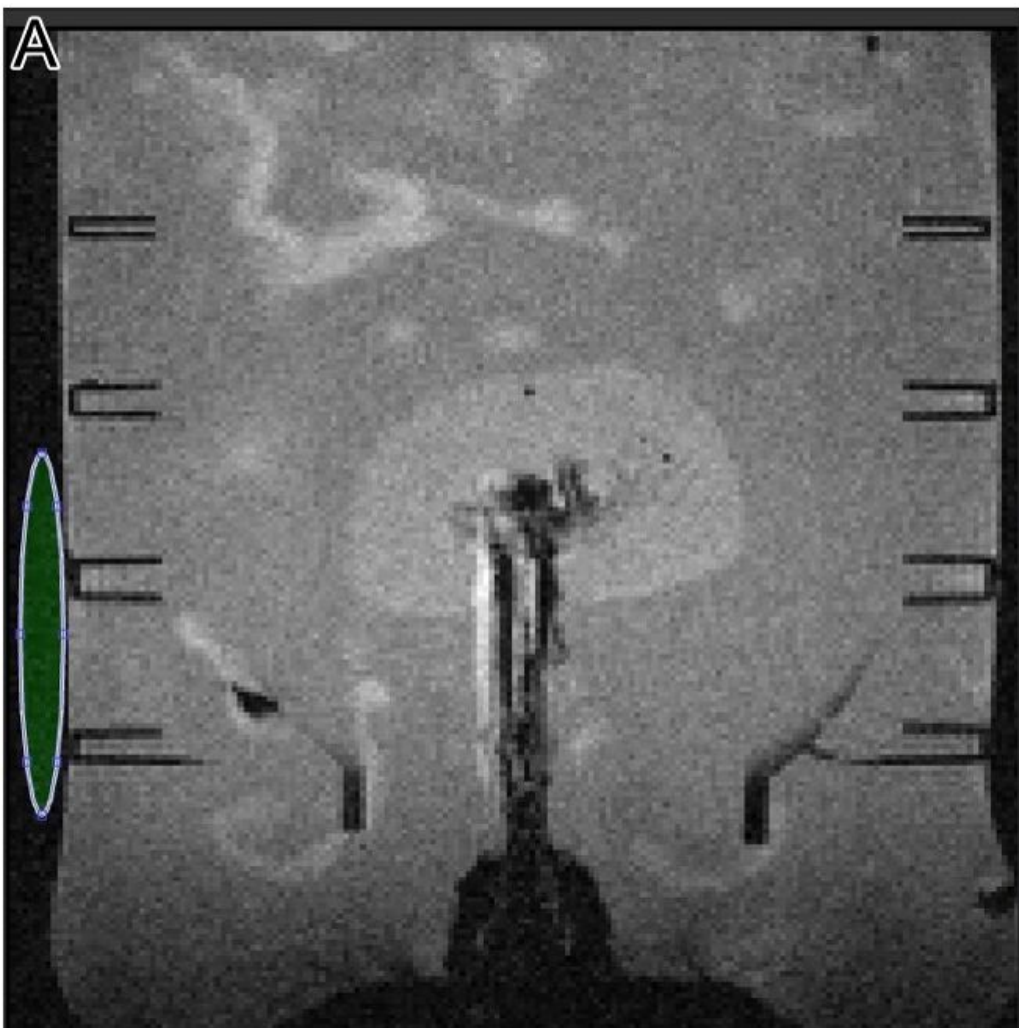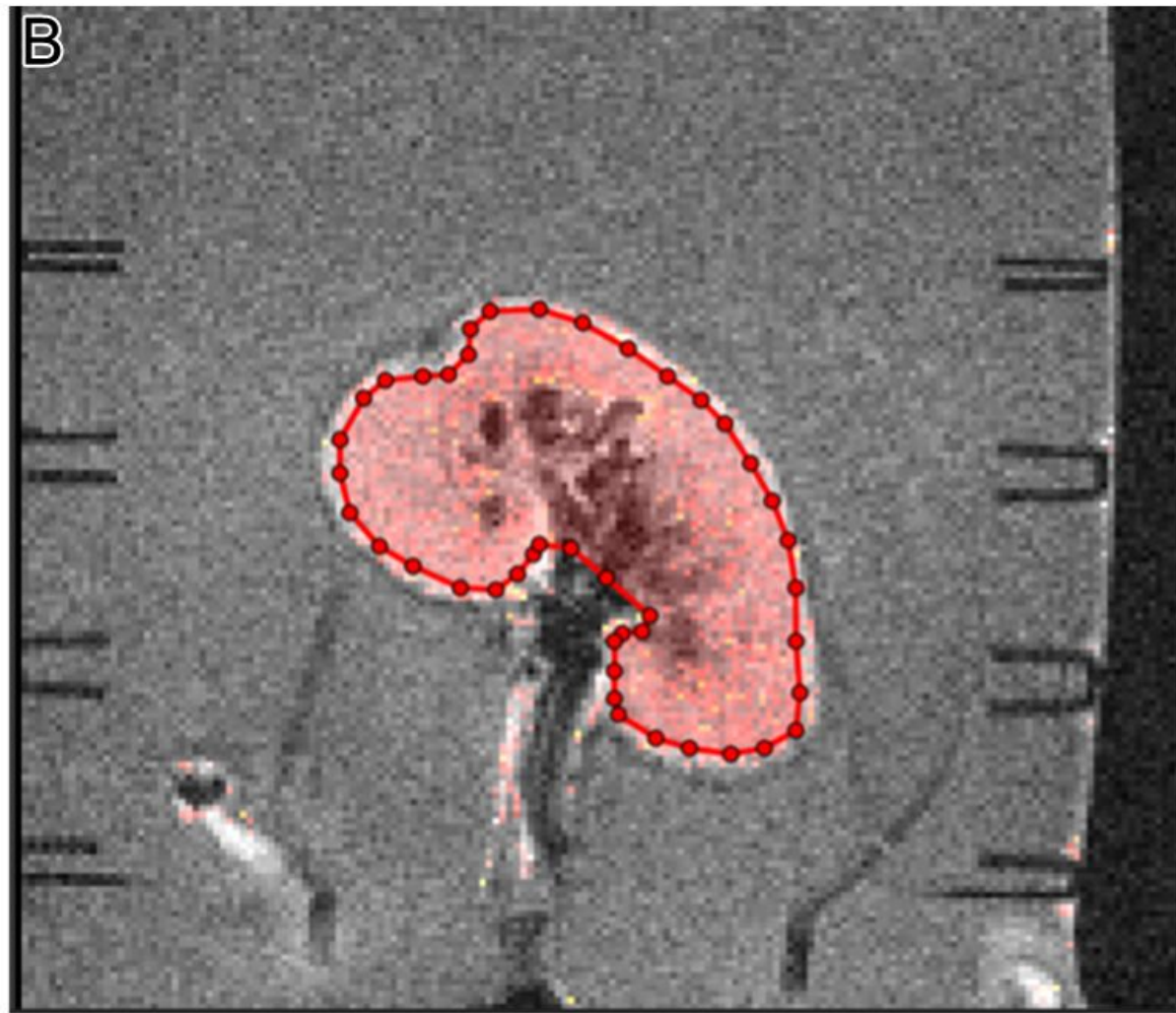

Supplement: Supplementary file 2 — Supplementary file2 Fig.2: A) Drawing an ROI (green) to determine the background noise in the area of air. B) Drawing an ROI (red) that includes only the renal parenchyma and renal pelvis. (PDF 146 KB) [file 345_2022_4255_MOESM2_ESM.pdf]

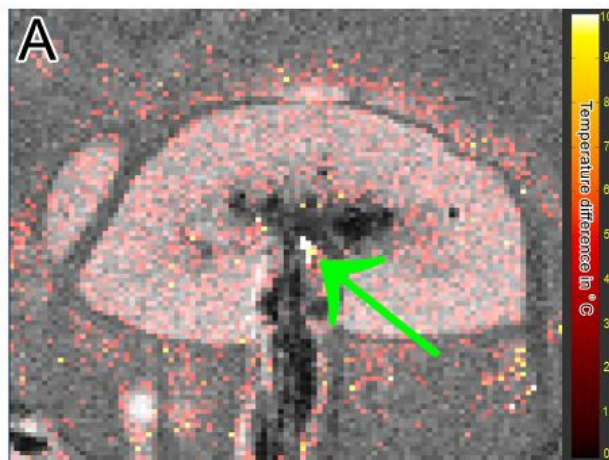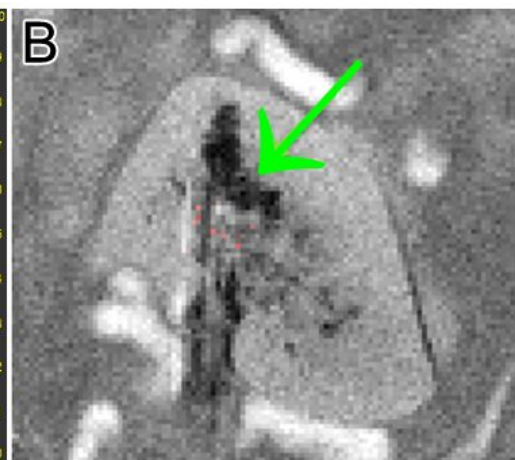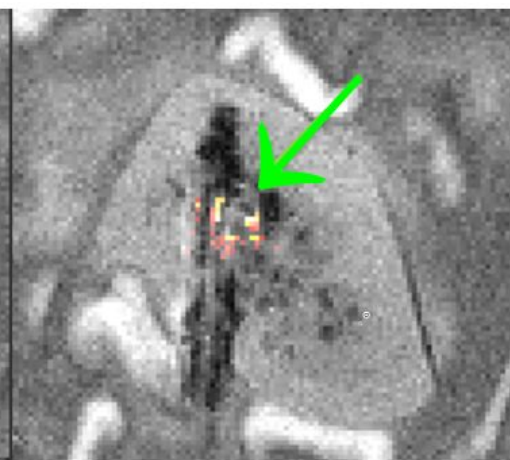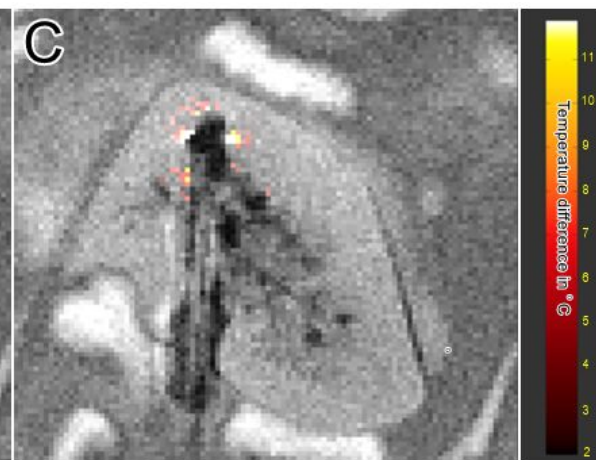

Supplement: Supplementary file 3 — Supplementary file3 Fig.3: A) Anatomical MR image (in gray) with superimposed temperature map (in glow scale) with a position of the fiber in the pelvis and an irrigation rate of 70 ml/min. At the tip of the laser fiber heating occurred at the edge of the renal pelvis near the hilum (green arrow). B) Lithotripsy with 14 W, tL=20 s and a flushing rate of 30 ml/min of stone granules in the calix before (left) and after (right) start of the laser application. C) Repeated lithotripsy with the same parameters as in B). (PDF 120 KB) [file 345_2022_4255_MOESM3_ESM.pdf]

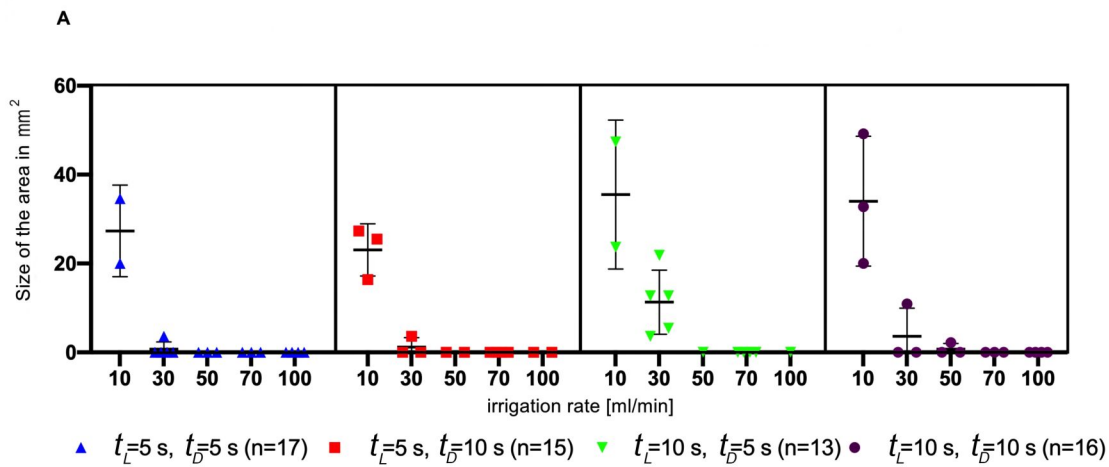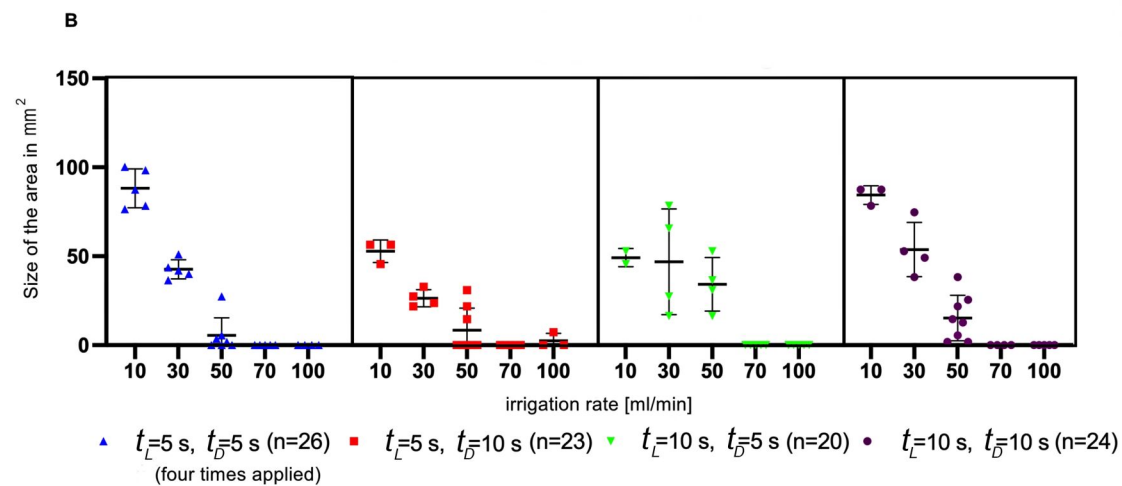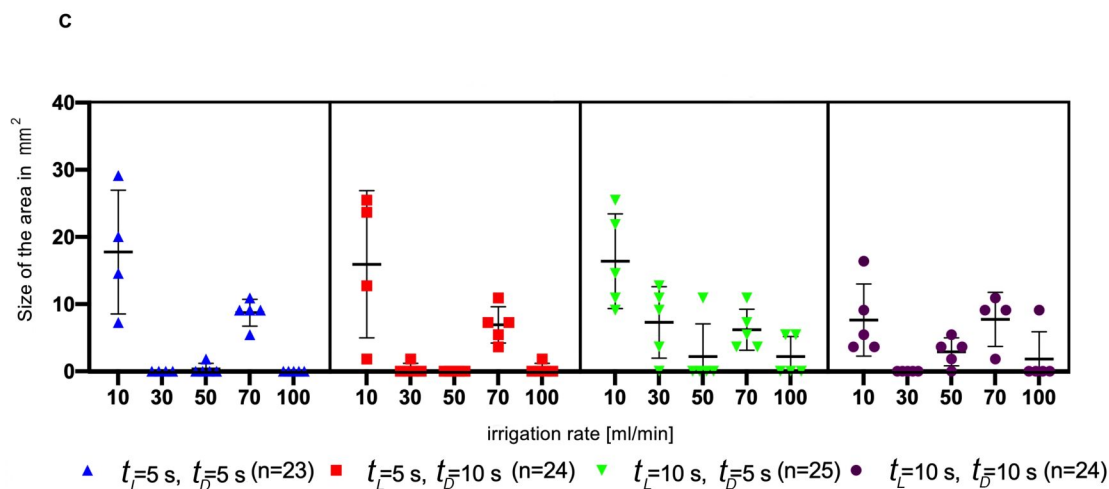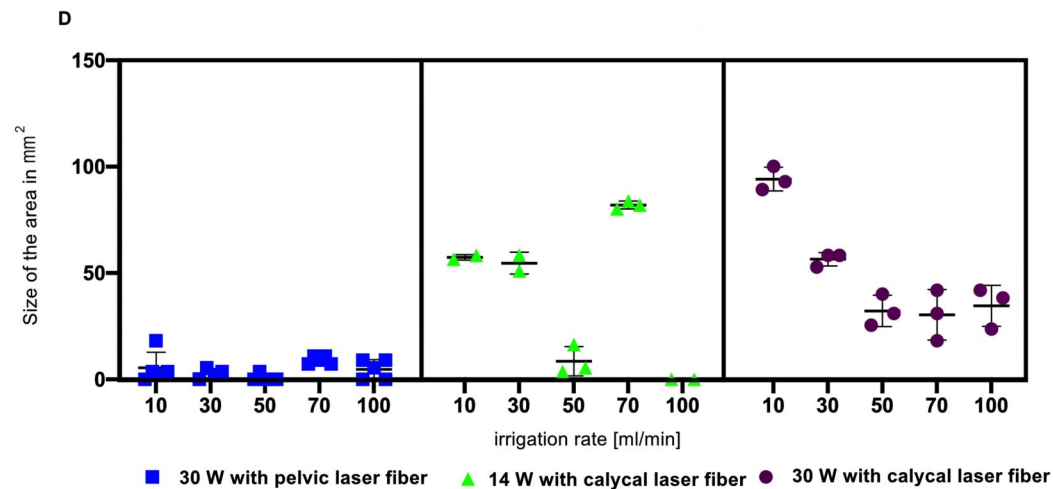

Supplement: Supplementary file 5 — Supplementary file5 Fig.4: Areas with CEM43> 120 min with different laser fiber positions and powers: A) 14 W [1.2 J, 12 Hz] and a fiber located in the renal calyx. B) 30 W [3.3 J, 9 Hz] and fiber located in the renal calyx. C) 30 W and fiber located in the renal pelvis. D) With tL=20 s. The mean values and the standard deviation of the respective test series are plotted in black. (PDF 240 KB) [file 345_2022_4255_MOESM5_ESM.pdf]

A

14 W with  $t_L = 20$  s,  $t_D = 0$  s

irrigation rate = 30 ml/min

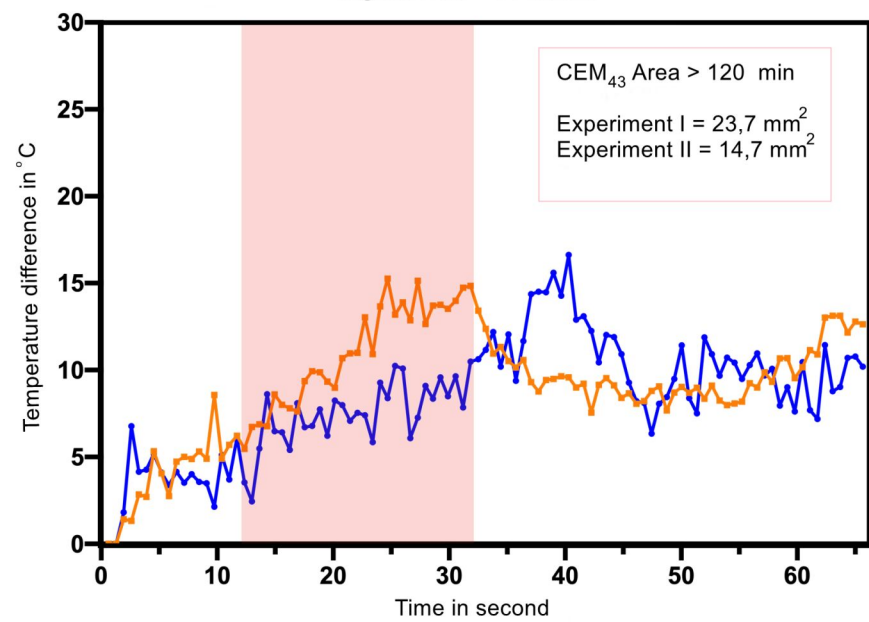

Experiment I  
Experiment II

B

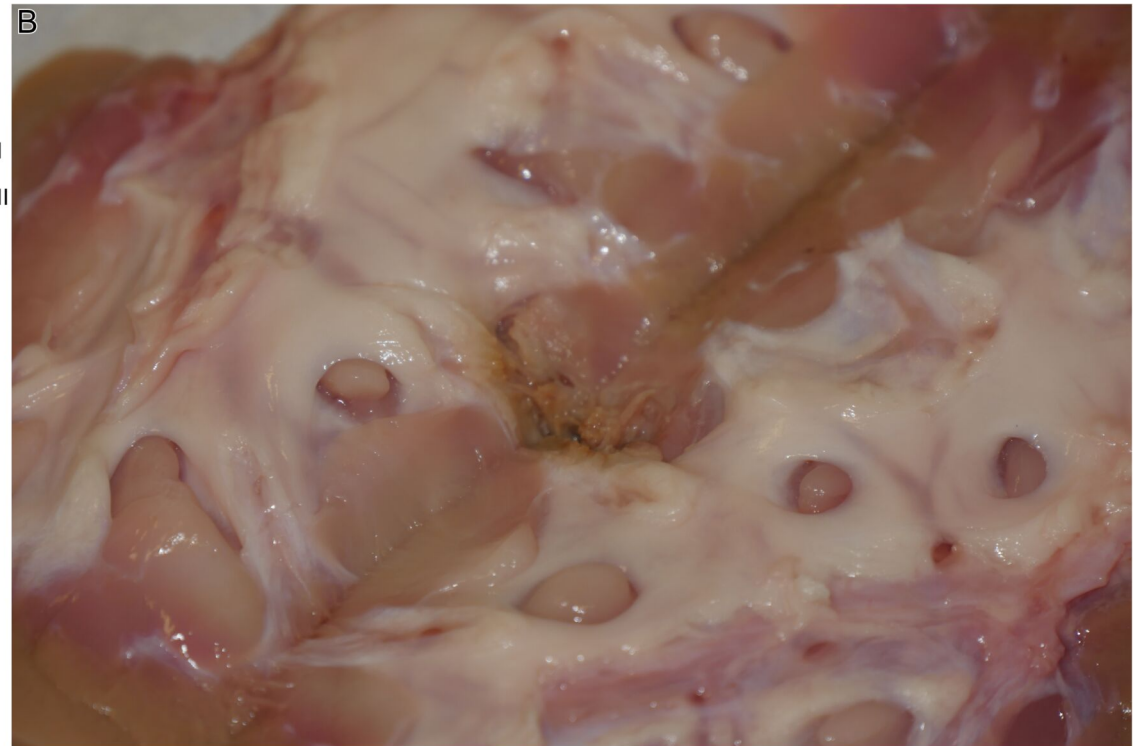

Supplement: Supplementary file 6 — Supplementary file6 Fig.5: A) Temperature curve during tL/tD=20/0 s with 14 W on stone granules in the kidney calyx and 30 ml/min flushing rate. The phase of the laser application is highlighted in red. B) Macroscopic view of the thermal lesion -tissue injury is clearly visible as a brown and dark area in the urothelium and the parenchyma. (PDF 204 KB) [file 345_2022_4255_MOESM6_ESM.pdf]
