# Supplementary material for: Assessing critical temperature dose areas in the kidney by magnetic resonance imaging thermometry in an ex vivo Holmium:YAG laser lithotripsy model
Source: World J Urol. 2022 Dec 21;41(2):543–9. doi: 10.1007/s00345-022-04255-1 (PMC9947089; doi:10.1007/s00345-022-04255-1)
Supplement: Supplementary file 4 — Supplementary file4 Table 1: Mean area of the pixels with CEM43> 120 min according to applied laser power and the irrigation rate. (PDF 42 KB) [file 345_2022_4255_MOESM4_ESM.pdf]

Table 1: Mean area of the pixels with CEM_{43}> 120 min according to applied laser power and the irrigation rate

			**Area with CEM_{43}> 120 min (mm^2)**				
**Laser power, kidney area**	**t_L/ t_D(n)**	***n***	***I* = 10 ml/min**	**30 ml/min**	**50 ml/min**	**70 ml/min**	**100 ml/min**
14 W, calyx	5 / 5	17	27.3	0.8	0	0	0
	5 / 10	15	23.1	1.2	0	0	0
	10 / 5	13	35.5	11.3	0	0	0
	10 / 10	16	34	3.6	0.7	0	0
30 W, calyx	5 / 5 (4 times)	26	88.2	42.6	5.5	0	0
	5 / 10	23	52.8	9.6	8.4	0	2.5
	10 / 5	20	49.1	47	34.1	0	0
	10 / 10	24	84.4	53.7	15.3	0	0
30 W, pelvis	5 / 5	23	17.75	0	0.4	8.7	0
	5 / 10	24	15.9	0.36	0	6.9	0.36
	10 / 5	25	16.39	7.28	2.18	6.19	2.18
	10 / 10	24	7.64	0	2.91	7.73	1.82

t_L: laser application time; t_D**:** delay time; *I:* irrigation rate**;** CEM_{43}: Cumulative Equivalent Minutes
